# Factors Associated With Entrepreneurial Intention Among Nursing Students: A Cross‐Sectional Study Using a Socio‐Ecological Model

**DOI:** 10.1002/nop2.70870

**Published:** 2026-10-06

**Authors:** Ji Young Lim, Wonjung Noh, Mohiniso Vohidova, Anna Lee

**Affiliations:** ^1^ School of Nursing, Inha University Incheon South Korea; ^2^ College of Nursing, Gachon University Incheon South Korea; ^3^ Department of Green Finance Inha University Incheon South Korea; ^4^ Mo‐Im Kim Nursing Research Institute, College of Nursing, Yonsei University Seoul South Korea; ^5^ Institute for Innovation in Digital Healthcare, Yonsei University Seoul South Korea

**Keywords:** Entrepreneurial intention, government support policies, nursing student, peer and family influence, socio‐ecological model

## Abstract

**Aim:**

To examine the factors associated with entrepreneurial intention among Korean nursing students using a socio‐ecological model.

**Design:**

A cross‐sectional study.

**Methods:**

This study, conducted in August 2024, involved 205 undergraduate nursing students who completed an online questionnaire assessing their entrepreneurial intention and individual, interpersonal, organizational, and policy‐level factors. Descriptive statistics and Pearson's correlation analyses were performed, followed by multiple linear regression analysis with entrepreneurial intention as the outcome variable and factors across the four socio‐ecological levels entered simultaneously as explanatory variables.

**Results:**

Peer influence and family influence were significantly associated with entrepreneurial intention, with family involvement being especially significant in Korea's collectivist culture. National economic conditions and government entrepreneurial support were also associated with students' entrepreneurial intention. Individual‐level characteristics were not significantly associated with entrepreneurial intention in the adjusted model.

**Conclusion:**

Nursing students' entrepreneurial intention was mainly associated with interpersonal and policy level factors. Nursing entrepreneurship education should strengthen peer and family support and increase awareness of government programs to prepare future nurses for innovation in evolving healthcare environments.

**Implications for the Profession and/or Patient Care:**

Nursing entrepreneurship education should extend beyond individual competency development by incorporating peer‐supported learning, culturally appropriate family engagement and practical information on government entrepreneurship support. These approaches may broaden professional career pathways and strengthen nurses' capacity to develop innovative healthcare services, technologies and care models.

**Impact:**

This study addressed the limited understanding of multilevel factors associated with entrepreneurial intention among nursing students. Interpersonal and policy‐level factors were more strongly associated with entrepreneurial intention than individual‐ or organizational‐level factors. The findings may inform nursing educators, educational institutions and policymakers seeking to strengthen entrepreneurship and innovation within the nursing profession.

**Reporting Method:**

This study was reported in accordance with the Strengthening the Reporting of Observational Studies in Epidemiology Statement for cross‐sectional studies.

**No Patient or Public Contribution:**

There was no involvement from patients or the public; the investigation exclusively examined nursing students' entrepreneurial intention and related determinants.

## 1 Introduction

The contemporary healthcare management environment is undergoing a profound transformation from a clinically centered model to a community‐oriented approach, with services becoming increasingly diversified and specialized to meet the evolving needs of diverse populations (Morris 2019). Navigating this shift requires healthcare professionals to possess advanced competencies, including organizational management, data analysis and financial administration. Yet existing educational programs often fall short in adequately preparing healthcare students—particularly in cultivating the entrepreneurial and leadership capacities necessary to navigate and shape this rapidly evolving landscape (Nkwanyana et al. 2023; Özyeşil et al. 2024). A recent scoping review found that healthcare science educational initiatives frequently neglect the development of entrepreneurial mindsets and the ability to generate innovative, healthcare‐specific business models (Suryavanshi et al. 2020). Nevertheless, longitudinal evidence suggests that entrepreneurial intention among healthcare students rises substantially—from 13% at graduation to 25.8% within five years (Sieger et al. 2024). These findings underscore the urgent need for curricular reforms that integrate structured training in entrepreneurship, leadership and strategic innovation, thereby equipping future healthcare professionals to address emerging opportunities and systemic challenges.

Within this evolving landscape, the nursing profession is uniquely positioned to drive innovation, leveraging its clinical expertise and central role in patient care. Nurses deliver patient‐centered care across prevention, treatment and rehabilitation, making them pivotal actors in advancing equitable healthcare access and spearheading technology‐driven solutions (Thepna et al. 2023; Jakobsen et al. 2021). Despite this potential, entrepreneurial engagement among nurses remains limited, with global participation estimated at less than 1% and negligible representation in countries such as South Korea (Jakobsen et al. 2021; Kim and Lim 2022). Strengthening the entrepreneurial capacity of nursing students is therefore both timely and strategically essential. In this study, “nursing students” refers to undergraduate, pre‐licensure students enrolled in nursing degree programs that prepare graduates for professional nursing practice and eligibility for registered nurse licensure.

Entrepreneurial intention refers to a purposeful mindset oriented toward creating a business, reflecting an individual's motivations, attitudes and perceived feasibility of entrepreneurship (Tsou et al. 2023). It constitutes a critical precursor to entrepreneurial behaviour, bridging the gap between aspiration and the concrete actions required to establish and sustain a venture (Antončič and Auer Antončič 2023). Researchers emphasize that entrepreneurship is inherently complex and uncertain, initiated and sustained by entrepreneurial intention (Kim and Lim 2022). Identifying the factors shaping entrepreneurial intention among students is therefore vital for promoting entrepreneurial activity within the nursing profession.

To understand the multiple factors influencing entrepreneurial intention, the socio‐ecological model provides a useful framework (Figure 1). It explains how individual behaviour is shaped by interacting influences across individual, interpersonal, organizational, community and societal levels (McLeroy et al. 1988). Applied to entrepreneurial intention, this model allows for a more comprehensive understanding of how personal characteristics and broader social and contextual factors jointly shape students' entrepreneurial decision‐making.

**FIGURE 1:**
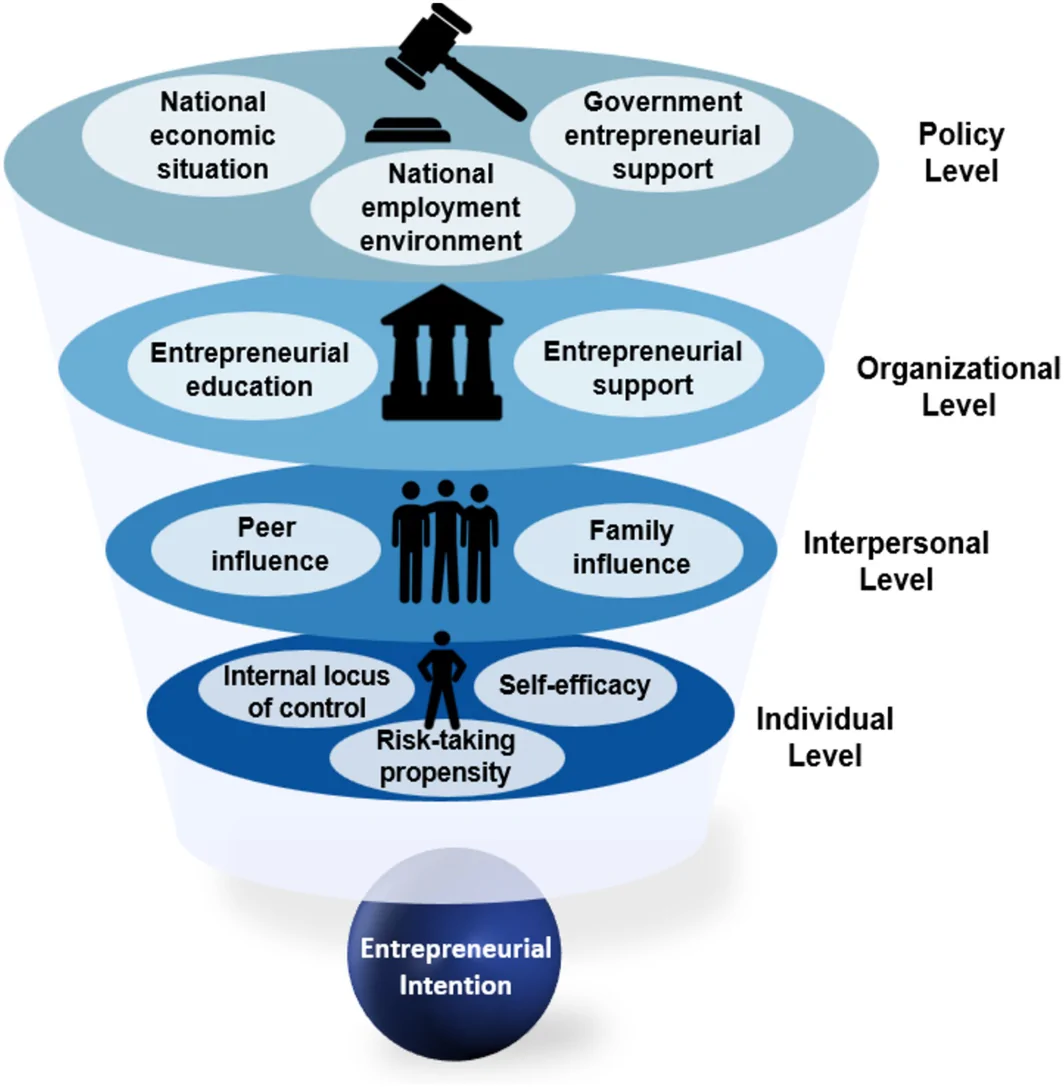
Socio‐ecological model used in this study. This figure was created by the authors based on the socio‐ecological model proposed by McLeroy et al. (1988).

At the individual level, empirical research demonstrates that entrepreneurial intention is influenced by individual traits such as locus of control, self‐efficacy, risk‐taking propensity and attitudes toward entrepreneurship (Antončič and Auer Antončič 2023; Cao et al. 2022). At the interpersonal level, family and peer support are equally important, with family business backgrounds enhancing entrepreneurial knowledge and competencies (Lingappa et al. 2020; Szabó and Aranyossy 2024). At the organizational and policy levels, macroeconomic, sociocultural and politico‐legal conditions also shape entrepreneurial intention, as taxation, education and infrastructure policies influence perceived opportunities (Global Entrepreneurship Monitor 2016; Organization for Economic Co‐operation and Development 2016). Moreover, government support and university‐based entrepreneurship programs can both stimulate entrepreneurial motivation and mitigate youth unemployment (Kim and Lim 2022; Zhang et al. 2022).

Although entrepreneurial intention has been widely studied in the general population, existing research has limitations that leave the research problem addressed in this study insufficiently understood. First, prior studies have predominantly examined individual‐level traits (e.g., self‐efficacy) in isolation, with comparatively few integrating individual, interpersonal, organizational and policy level factors within a single explanatory model (Tetteh et al. 2024). This limitation underscores the need for a socio‐ecological perspective to better understand entrepreneurial intention.

Second, comparatively little attention has been directed toward nursing students within this multilevel perspective, despite their unique potential to drive innovation within healthcare systems (Gardim et al. 2024). This gap is particularly salient in Korea, where collectivist cultural norms and structural economic conditions exert a strong influence on career trajectories and professional decision‐making.

To address this gap, the present study investigates the individual, interpersonal, organizational and policy level factors associated with entrepreneurial intention among Korean nursing students. Guided by the socio‐ecological model, the study aims to generate actionable insights to enhance entrepreneurship education and inform healthcare policy development. The research questions were as follows:

(1) What are the levels of entrepreneurial intention and related individual, interpersonal, organizational and policy level factors among Korean nursing students?

(2) Which individual, interpersonal, organizational and policy level factors are associated with entrepreneurial intention among Korean nursing students?

## 2 Methods

### 2.1 Design

This cross‐sectional study aimed to identify the factors associated with entrepreneurial intention among Korean nursing students using a socio‐ecological model (McLeroy et al. 1988). The specific objectives were to assess the levels of entrepreneurial intention and its related factors, and to examine associations between entrepreneurial intention and individual, interpersonal, organizational and policy level factors among Korean nursing students. This manuscript was prepared and reported in accordance with the Strengthening the Reporting of Observational Studies in Epidemiology Statement for cross‐sectional studies.

### 2.2 Sample and Data Collection

Data were collected in August 2024 using Google Forms. Eligible participants were undergraduate nursing students enrolled in Korean nursing degree programs designed to prepare graduates for professional nursing practice and eligibility for registered nurse licensure. Individuals unable to complete the online survey were excluded. As of 2024, the total population comprised 52,301 students enrolled in 129 nursing degree programs nationwide (Korean Educational Development Institute 2024). Because participants were recruited through closed online nursing student communities rather than a national sampling frame, the exact size of the accessible population could not be determined. A sample size of 172 was calculated using G*Power 3.1.9.7 for multiple regression analysis, based on an alpha level of 0.05, a medium effect size of 0.15, a power of 0.95 and 10 explanatory variables. Given our actual sample size of 205, it was deemed sufficient for reliable analysis.

Participants were recruited using a convenience sampling strategy through an online flyer posted in closed online communities exclusively accessible to nursing students. These platforms included students across various academic years and institutional types (e.g., public and private universities), capturing a broad cross‐section of the undergraduate nursing student population. Prior to posting, the researcher obtained permission from community administrators by providing a detailed explanation of the study's objectives and procedures. The recruitment flyer, which included a QR code linking to the online survey, was uploaded as a non‐editable PDF and remained available for two weeks.

The online survey included an introduction to the study, an informed consent form, a screening question to confirm eligibility, and items measuring general characteristics, entrepreneurial intention and variables at the individual, interpersonal, organizational and policy levels. All survey items were designated as mandatory within the online platform to minimize missing data.

### 2.3 Measurements

The researchers obtained permission to use all measurement instruments and translated those originally developed in English into Korean with the original authors' approval. Translation and cultural adaptation were conducted in accordance with the guidelines proposed by Sousa and Rojjanasrirat (2011). A forward–backward translation procedure was conducted by bilingual experts to ensure linguistic and conceptual equivalence. Discrepancies were resolved through research team consensus, and minor revisions were made for clarity and cultural relevance. Content validity was evaluated using the Content Validity Index (CVI) by four experts in nursing management, entrepreneurship and business studies. All items achieved a CVI above 0.80, indicating satisfactory content validity (Lynn 1986). After data collection, confirmatory factor analysis (CFA) was conducted to further evaluate the translated instruments.

#### 2.3.1 Entrepreneurial Intention

To examine the nursing students' entrepreneurial intention, this study adopted the Korean version of the Entrepreneurial Intention Questionnaire originally developed by Liñán and Chen (2009), following the five‐item operationalization used by Jaén and Liñán (2013). This scale consists of five items rated on a seven‐point scale, with higher scores indicating stronger entrepreneurial intention. However, based on the results of a CFA, one item with an insufficient factor loading was excluded, resulting in a final scale of four items. One sample item is “I have a strong determination to start my own business in the future.” The reliability of the scale was confirmed with a Cronbach's α of 0.94 (Liñán and Chen 2009) and 0.92 in this study.

#### 2.3.2 Individual Level

##### 2.3.2.1 Entrepreneurial Self‐Efficacy

To assess entrepreneurial self‐efficacy, we adopted the Korean version of the Entrepreneurial Self‐Efficacy Scale developed by Wilson et al. (2007). This scale consists of six items rated on a five‐point scale, with higher scores indicating greater entrepreneurial self‐efficacy. Based on the results of a CFA, two items with insufficient factor loadings were excluded, resulting in a final scale of four items. One sample item is “Compared with other students, my leadership ability is high.” The scale demonstrated a Cronbach's α of 0.82 for business school graduates (Wilson et al. 2007) and 0.74 in this study.

##### 2.3.2.2 Internal Locus of Control

To examine the internal locus of control, the study used eight items related to internal control from Spector's Locus of Control Scale (Spector 1988), translated into Korean. These items are rated on a six‐point scale, with higher scores indicating a greater internal locus of control. One sample item is “Knowing what I want from a career helps me find a career that meets my expectations.” In a previous study, the scale demonstrated a Cronbach's α of 0.79 (Song 2022); in this study, it yielded a value of 0.78.

##### 2.3.2.3 Risk‐Taking Propensity

The Risk‐Taking Propensity Scale developed by Lüthje and Franke (2003) was translated into Korean to measure the risk‐taking propensity. This scale consists of three items rated on a five‐point scale, with higher scores indicating greater risk‐taking propensity. One sample item is “I have engaged in risk‐taking behaviours during the past six months.” In a previous study, the scale showed a Cronbach's α of 0.62 (Lüthje and Franke 2003); it was 0.72 in this study.

#### 2.3.3 Interpersonal Level

##### 2.3.3.1 Peer Influence

To measure peer influence on entrepreneurship, this study utilized the Peer Influence Scale developed by Lingappa et al. (2020) and translated into Korean. The scale consists of four items rated on a seven‐point scale, with higher scores indicating stronger peer influence on entrepreneurship. One sample item is “I have friends who value entrepreneurship.” In previous research, the scale demonstrated a Cronbach's α of 0.74 (Lingappa et al. 2020); in this study, it was 0.84.

##### 2.3.3.2 Family Influence

To measure family influence on entrepreneurship, this study employed the Family Influence Scale developed by Lingappa et al. (2020) and translated into Korean. This scale consists of three items rated on a seven‐point scale, with higher scores indicating stronger family influence on entrepreneurship. One sample item is “I currently have a self‐employed family member.” In previous research, the scale demonstrated a Cronbach's α of 0.60 (Lingappa et al. 2020); in this study, it was 0.66.

#### 2.3.4 Organizational Level

##### 2.3.4.1 Entrepreneurial Education at School

To measure the perceived level of entrepreneurial education in school among nursing students, this study used the School Entrepreneurial Education Scale developed by Lingappa et al. (2020), translated into Korean. This scale comprises four items rated on a seven‐point scale, with higher scores indicating a higher level of perceived entrepreneurial education at school. One sample item is “My university provides education on business management concepts.” The scale demonstrated a Cronbach's α of 0.73 in previous research (Lingappa et al. 2020) and 0.79 in this study.

##### 2.3.4.2 Entrepreneurial Support at School

To measure the perceived level of entrepreneurial support at school among nursing students, this study employed the School Entrepreneurial Support Scale developed by Lingappa et al. (2020), translated into Korean. This scale consists of five items rated on a seven‐point scale, with higher scores indicating a higher level of perceived entrepreneurial support at school. One sample item is “My university has adequate infrastructure to support entrepreneurial activities.” The scale demonstrated a Cronbach's α of 0.83 in previous research (Lingappa et al. 2020) and 0.91 in this study.

#### 2.3.5 Policy Level

##### 2.3.5.1 National Economic Situation

Hwang's (2020) Economic Situation Scale was used to measure nursing students' perceptions of the national economic situation. This scale includes five items rated on a five‐point scale. Higher scores indicate a more positive perception of the economic situation. One sample item is “I believe that the current national economic conditions are favourable.” The scale demonstrated a Cronbach's α of 0.75 in previous research (Hwang 2020) and 0.77 in this study.

##### 2.3.5.2 National Employment Environment

To measure nursing students' perceptions of the national employment environment, the study used the Employment Environment Scale developed by Hwang (2020). This scale includes five items rated on a five‐point scale. Higher scores indicate a more negative environment for employment. Based on the results of a CFA, one item with an insufficient factor loading was excluded, resulting in a final scale of four items. One sample item is “I perceive the current national employment situation negatively.” The scale demonstrated a Cronbach's α of 0.80 in previous research (Hwang 2020) and 0.80 in this study.

##### 2.3.5.3 Government Entrepreneurial Support

To measure nursing students' perceptions of national entrepreneurial support systems, this study used the Entrepreneurial Support Policy Scale developed by Park and Nam (2022). The scale consists of eight items rated on a five‐point Likert scale, with higher scores reflecting a greater perceived level of national entrepreneurial support. One sample item is “I am familiar with government policies that support entrepreneurship education, such as entrepreneurship courses and entrepreneurship academies.” The scale demonstrated a high reliability with a Cronbach's α of 0.98 in previous research (Park and Nam 2022) and 0.94 in this study.

### 2.4 Data Analysis

Data were analysed using IBM SPSS Statistics for Windows, Version 31.0 (IBM Corp 2025). Descriptive statistics were used to summarize participants' characteristics and major study variables. Prior to analysis, the dataset was screened for completeness, validity and plausibility. Because all survey items were designated as mandatory, there were no missing data for the primary study variables. One participant entered nonnumeric text for age; this response was treated as missing for age only, while the participant's remaining data were retained. Accordingly, age was summarized using data from 204 participants, whereas all primary analyses were conducted using the full sample of 205 participants. No data were imputed. No sensitivity analysis was performed because age was not included in the primary correlation or regression analyses.

Scale scores were calculated according to each instrument's scoring procedure and treated as continuous variables. As part of the psychometric evaluation of the translated instruments, CFA was conducted using the lavaan package in R via Google Colaboratory. Items with standardized factor loadings below 0.40 were removed from the relevant scales (Hair et al. 2010), and final scale scores were calculated using the retained items. Standardized factor loadings, average variance extracted, composite reliability and Cronbach's alpha were then recalculated for the refined scales. All descriptive, correlation and regression analyses were conducted using the refined scale scores.

Pearson's correlation coefficients were calculated to examine bivariate associations between entrepreneurial intention and individual, interpersonal, organizational and policy level variables. *p*‐Values were calculated using two‐tailed Pearson's correlation analysis. Multiple linear regression analysis was conducted with entrepreneurial intention as the outcome variable and all individual, interpersonal, organizational and policy level variables entered simultaneously as explanatory variables. The *p*‐value for the overall regression model was based on the *F*‐test, and *p*‐values for individual explanatory variables were based on *t*‐tests of regression coefficients.

Regression assumptions were examined by identifying extreme outliers (Studentized residuals beyond ±3) and evaluating normality using the Shapiro–Wilk test. Multicollinearity was not a concern, as all tolerance values exceeded 0.10 (Polit 2010). Exact *p*‐values were reported in the Results narrative and regression table, except for values smaller than 0.001, which were reported as *p* < 0.001. For the correlation matrix, significance levels were indicated using footnote symbols for readability.

### 2.5 Ethical Considerations

The study followed the principles of the Declaration of Helsinki and was approved by the Yonsei University Health System Institutional Review Board (IRB no. 4–2024‐0561). Prior to beginning the online survey, participants were presented with an informed consent statement on the first page of the Google Forms questionnaire, which described the study's purpose, the voluntary nature of participation, the confidentiality of responses and participants' right to withdraw. Participants were informed that they could discontinue the survey at any time before submission by closing the browser window, and that declining to participate or withdrawing would involve no penalty or disadvantage. Only those who indicated informed consent were permitted to proceed with the survey items. Participants who agreed to the study's purpose voluntarily participated in it, and upon completing the online survey, they received a mobile coupon valued at approximately 4 USD.

To protect confidentiality and anonymity, the survey did not collect directly identifying information (e.g., names, student IDs and email addresses), precluding identifier‐based duplicate detection. Duplicate participation was instead minimized through explicit single‐response instructions, an eligibility screening question and post hoc review of the dataset for implausible or duplicate response patterns. Response data were stored in a password‐protected file accessible only to the research team, analysed in de‐identified form and reported only in aggregate.

## 3 Results

### 3.1 Sample Characteristics and Major Variables

A total of 205 nursing students participated in this study, with a mean age of 21.97 years (SD = 2.21); most were female (*n* = 185, 90.2%). Seniors constituted the largest proportion (*n* = 83, 40.5%) (Table 1). Most participants had prior work experience (*n* = 159, 77.6%), whereas only 2.4% (*n* = 5) had prior start‐up experience.

**TABLE 1:** Sample Characteristics (*N* = 205).

Variable	*n* (%)	Mean (SD)
Age (years)^a^		21.97 (2.21)
Gender		
Male	20 (9.8)	
Female	185 (90.2)	
Grade		
1st year student	23 (11.2)	
2nd year student	47 (22.9)	
3rd year student	52 (25.4)	
4th year student	83 (40.5)	
Work experience		
Yes	159 (77.6)	
No	46 (22.4)	
Start‐up experience		
Yes	5 (2.4)	
No	200 (97.6)	

Abbreviation: SD, standard deviation.

^a^ Age was analysed using valid data from 204 participants because one participant entered nonnumeric text rather than a valid numeric age. All other variables were analysed using the full sample (*N* = 205).

Table 2 summarizes entrepreneurial intention and related socio‐ecological factors. The mean entrepreneurial intention score was 12.77 (SD = 6.03), corresponding to an item‐level mean of 3.19 on the 7‐point scale, which was below the item‐level scale midpoint of 4.00. Because the related variables were measured using instruments with differing item numbers and scoring ranges, their descriptive statistics are interpretable only within, not across, constructs.

**TABLE 2:** Major Variables (*N* = 205).

Variables	Min	Max	Mean (SD)	Loading range	AVE	CR
Entrepreneurial intention	4	28	12.77 (6.03)	0.841–0.873	0.733	0.917
Individual level						
Self‐efficacy	6	20	15.45 (2.55)	0.490–0.722	0.426	0.744
Internal locus of control	18	48	36.59 (5.12)	0.499–0.633	0.312	0.783
Risk‐taking propensity	3	15	9.73 (2.89)	0.524–0.796	0.499	0.744
Interpersonal level						
Peer influence	4	28	13.70 (6.46)	0.613–0.829	0.595	0.853
Family influence	3	21	10.29 (5.29)	0.455–0.863	0.443	0.692
Organizational level						
Entrepreneurial education at school	4	26	15.50 (5.17)	0.492–0.932	0.560	0.827
Entrepreneurial support at school	5	35	17.73 (7.06)	0.758–0.863	0.662	0.907
Policy level						
National economic situation	5	21	11.35 (3.69)	0.576–0.734	0.419	0.782
National employment environment	4	17	7.93 (3.27)	0.613–0.889	0.537	0.820
Government entrepreneurial support	8	38	15.27 (6.76)	0.747–0.870	0.654	0.938

Abbreviations: AVE, average variance extracted; CR, composite reliability; SD, standard deviation.

### 3.2 Relationships Between Entrepreneurial Intention and Factors at Each Level

Entrepreneurial intention showed a strong positive correlation with peer influence (*r* = 0.569, *p* < 0.001) (Table 3). Moderate positive correlations were observed with family influence (*r* = 0.419, *p* < 0.001) and government entrepreneurial support (*r* = 0.368, *p* < 0.001). Weak positive correlations were found with self‐efficacy (*r* = 0.205, *p* = 0.003), risk‐taking propensity (*r* = 0.295, *p* < 0.001), internal locus of control (*r* = 0.283, *p* < 0.001), entrepreneurial education at school (*r* = 0.227, *p* = 0.001), and entrepreneurial support at school (*r* = 0.205, *p* = 0.003). A weak negative correlation was observed with perceived national economic situation (*r* = −0.151, *p* = 0.031), whereas national employment environment was not significantly correlated with entrepreneurial intention (*r* = −0.056, *p* = 0.422).

**TABLE 3:** Correlations Among Major Variables.

	Entreprene‐urial intention	Self‐efficacy	Internal locus of control	Risk‐taking propensity	Peer influence	Family influence	Entreprene‐urial education	Entreprene‐urial support	Economic situation	Employment environment	Government support
Entrepreneurial intention	—										
Self‐efficacy	0.205**	—									
Internal locus of control	0.283***	0.437***	—								
Risk‐taking propensity	0.295***	0.248***	0.201**	—							
Peer influence	0.569***	0.223**	0.306***	0.418***	—						
Family influence	0.419***	0.077	0.253***	0.269***	0.383***	—					
Entrepreneurial education	0.227**	0.120	0.216**	0.185**	0.298***	0.281***	—				
Entrepreneurial support	0.205**	0.046	0.083	0.197**	0.297***	0.146*	0.557***	—			
Economic situation	−0.151*	0.008	−0.026	−0.163*	−0.071	−0.146*	−0.033	0.118	—		
Employment environment	−0.056	0.026	0.007	−0.169*	−0.163*	−0.148*	−0.208**	−0.105	0.413***	—	
Government support	0.368***	0.238***	0.089	0.158*	0.267***	0.068	0.154*	0.237***	0.145*	0.057	—

*Note:* *p < 0.05, **p < 0.01, ***p < 0.001.

### 3.3 Factors Associated With Entrepreneurial Intention

The overall regression model was statistically significant (*F* = 16.18, *p* < 0.001) and explained 45% of the variance in entrepreneurial intention (*R* ^2^ = 0.45, adjusted *R* ^2^ = 0.43), indicating that the included individual, interpersonal, organizational and policy level factors accounted for a substantial proportion of variability in entrepreneurial intention (Table 4).

**TABLE 4:** Multiple Regression Analysis of Factors Associated With Entrepreneurial Intention.

Variable	*t*	*p*	*β*	95% CI for B	Partial *f* ^2^	VIF
Individual level						
Self‐efficacy	0.07	0.941	0.00	[−0.276, 0.298]	0.000	1.35
Internal locus of control	1.23	0.219	0.08	[−0.054, 0.235]	0.008	1.38
Risk‐taking propensity	0.02	0.983	0.00	[−0.248, 0.254]	0.000	1.32
Interpersonal level						
Peer influence	5.95	< 0.001	0.39	[0.244, 0.486]	0.183	1.53
Family influence	3.65	< 0.001	0.22	[0.115, 0.385]	0.069	1.28
Organizational level						
Entrepreneurial education at school	−0.10	0.921	−0.01	[−0.163, 0.148]	0.000	1.63
Entrepreneurial support at school	0.34	0.736	0.02	[−0.094, 0.132]	0.001	1.60
Policy level						
National economic situation	−2.78	0.006	−0.17	[−0.470, −0.080]	0.040	1.30
National employment environment	1.57	0.117	0.10	[−0.044, 0.395]	0.013	1.30
Government entrepreneurial support	4.42	< 0.001	0.26	[0.126, 0.330]	0.101	1.19

*Note: F*(10, 194) = 16.18, *p* < 0.001, *R* ^2^ = 0.45, Adj *R* ^2^ = 0.43, Durbin‐Watson = 1.85. Partial *f* ^2^ was interpreted as negligible (< 0.02), small (0.02–0.14), medium (0.15–0.34) and large (≥ 0.35).

Abbreviations: CI, confidence interval; VIF, variance inflation factor.

Interpreted within the socio‐ecological framework, interpersonal level factors were the most prominent explanatory variables. Peer influence was the strongest positive explanatory variable of entrepreneurial intention (*β* = 0.39, *p* < 0.001), with a medium effect size (partial *f* ^2^ = 0.183). Family influence was also a significant positive explanatory variable (*β* = 0.22, *p* < 0.001), with a small effect size (partial *f* ^2^ = 0.069).

At the policy level, government entrepreneurial support was a significant positive explanatory variable of entrepreneurial intention (*β* = 0.26, *p* < 0.001), with a small effect size (partial *f* ^2^ = 0.101). National economic situation was a significant negative explanatory variable (*β* = −0.17, *p* = 0.006), with a small effect size (partial *f* ^2^ = 0.040), indicating that students who perceived the national economic situation more positively tended to report lower entrepreneurial intention after adjusting for other variables.

In contrast, individual level factors, including self‐efficacy, internal locus of control and risk‐taking propensity; organizational level factors, including entrepreneurial education and support at school; and national employment environment were not significant explanatory variables in the adjusted model.

## 4 Discussion

This study explored the factors associated with nursing students' entrepreneurial intention through the lens of the socio‐ecological framework. This study examined two research questions: first, the levels of entrepreneurial intention and related individual, interpersonal, organizational and policy level factors among Korean nursing students; and second, which socio‐ecological factors were associated with entrepreneurial intention.

Regarding the first research question, nursing students reported a lower level of entrepreneurial intention than in other studies using similar 7‐point entrepreneurial intention measures, while the levels of related factors varied across the socio‐ecological domains. Using a refined four‐item version of the Entrepreneurial Intention Questionnaire (Liñán and Chen 2009; adapted from Jaén and Liñán 2013), the current study's item‐level mean was 3.19, lower than the means reported by Wu et al. (2019) using the original five‐item instrument among MBA students in China (4.34) and by Molino et al. (2018) among Italian samples (5.16 for men, 4.00 for women). Because the present study used a refined four‐item version following CFA‐based item removal, whereas Wu et al. (2019) and Molino et al. (2018) used the original five‐item version, direct comparisons should be interpreted cautiously, particularly given further differences in participant characteristics, academic disciplines and cultural contexts. In addition to these measurement differences, which alone could account for part of the observed gap, the comparatively low score may also reflect Korean nursing students' stronger orientation toward stable hospital‐based employment and their limited exposure to nursing entrepreneurship. These findings suggest a need for nursing education to strengthen students' entrepreneurial intention.

Regarding the second research question, the findings showed that entrepreneurial intention was more strongly associated with interpersonal and policy level factors than with individual or organizational level factors. These findings advance understanding of entrepreneurial intention among nursing students, highlighting the interplay among multiple factors.

Specifically, at the individual level, self‐efficacy, internal locus of control and risk‐taking propensity were not significant explanatory variables of entrepreneurial intention in the adjusted model. This finding should not be interpreted as indicating that individual characteristics are irrelevant to entrepreneurial intention. Rather, it suggests that their explanatory contribution may have been attenuated after interpersonal, organizational and policy level factors were considered simultaneously. While recent international studies have emphasized personal characteristics as primary drivers of entrepreneurial behaviour (Antončič and Auer Antončič 2023; Karimi et al. 2017), the present findings suggest that among Korean nursing students, entrepreneurial intention may be shaped more strongly by social and structural conditions, shifting attention to interpersonal and structural factors.

One plausible structural explanation is that nursing students' career decisions are guided less by individual dispositions than by the perceived stability of the profession itself. This result is particularly pertinent within the nursing field, where students often prioritize stable career paths over entrepreneurial ventures due to the perceived security of traditional roles (Ait Ali et al. 2024). Recognizing this, educators and policymakers should broaden the spectrum of career pathways in nursing, illustrating how entrepreneurial competencies can be leveraged to address emerging healthcare challenges such as population aging and the growing burden of chronic diseases. By framing entrepreneurship as a complementary rather than an alternative career path, students may feel more empowered to explore entrepreneurial ventures.

At this same individual level, demographic characteristics such as gender also warrant consideB34ration alongside the psychological traits examined above, even though gender was not entered as an explanatory variable in the regression model. In this study, female students comprised 90.2% of participants, reflecting the historically female‐dominated composition of nursing education and the nursing workforce (Pincha Baduge et al. 2024). This gender profile may be particularly relevant to nursing entrepreneurship since women may encounter distinct sociocultural expectations and barriers related to professional networks, financial resources and career advancement (Pincha Baduge et al. 2024; Rejeb et al. 2026). Nursing entrepreneurship education should therefore address the professional and sociocultural contexts experienced by women in nursing while remaining inclusive of students of all genders.

At the interpersonal level, peer influence was the strongest explanatory variable of entrepreneurial intention. This finding is consistent with prior research showing that peers can shape entrepreneurial attitudes, perceived behavioural control and social norms. Lingappa et al. (2020), in a survey of 210 undergraduate engineering students, identified a significant association between peer influence and entrepreneurial attitudes, perceived behavioural control and social norms. Similarly, Bellò et al. (2018) found that peers who actively promoted entrepreneurship moderated the relationship between creativity and entrepreneurial intention among 507 individuals aged 17–21.

Portyanko et al. (2023) elucidated two mechanisms through which peers shape entrepreneurial intention: information and approval. Information mechanisms involve acquiring and evaluating peer‐provided insights such as informal advice that help refine business ideas, expand entrepreneurial knowledge, reduce uncertainty and guide decision‐making. Approval mechanisms reflect perceived social endorsement, whereby positive peer attitudes reinforce commitment and motivation toward entrepreneurship. This process mirrors the subjective norm in Ajzen's (1991) theory of planned behaviour, which emphasizes the role of perceived peer expectations in shaping intention. Together, these mechanisms provide a framework for understanding how peer influence fosters entrepreneurial orientation among nursing undergraduates.

Given that nursing students are typically in a developmental stage where peer influence is particularly salient, and considering the collaborative nature of nursing education through clinical practice and group work, such influence may be amplified. Nurse educators should therefore acknowledge peers as a critical driver of entrepreneurial intention and foster both knowledge‐sharing and peer‐supported learning environments to enhance entrepreneurial motivation in nursing education.

In this study, family influence also emerged as a significant factor, consistent with recent international studies showing that family support and family business background can shape students' entrepreneurial intention (Lingappa et al. 2020; Szabó and Aranyossy 2024; Tiwari et al. 2020). In collectivist societies such as Korea, familial influence is particularly salient (Lee et al. 2022). Entrepreneurial education for nursing students in such contexts may benefit from incorporating family engagement—through informational sessions or discussions that align entrepreneurial pursuits with collectivist values and community benefits.

Organizational level factors, including entrepreneurial education and support at school, were not significant explanatory variables in the adjusted model. This does not necessarily indicate that school‐based entrepreneurship education is unimportant; rather, existing educational exposure or institutional support may not yet be sufficiently visible, accessible, or tailored to nursing students' professional contexts. Nursing entrepreneurship education may therefore need to move beyond general business concepts and explicitly connect entrepreneurship to nursing roles, clinical innovation, digital health and community‐based care, framing it as a complementary professional competency that helps students address emerging healthcare needs such as population aging, chronic disease management and technology‐enabled care (Wang et al. 2024).

At the policy level, a particularly counterintuitive finding emerged: students who perceived the national economic environment more positively reported lower entrepreneurial intention. This finding challenges the traditional view that favourable economic conditions stimulate entrepreneurship (Paulsen et al. 2012; University of Bristol 2019). However, evidence indicates that robust economies with abundant job opportunities can instead reduce entrepreneurial intention, as youth may prioritize secure employment over entrepreneurial risk (Xanthopoulou and Sahinidis 2024). In Korea, this effect is amplified by a competitive job market and a cultural preference for stable roles in established sectors such as healthcare (Organization for Economic Co‐operation and Development 2024).

Perceived government entrepreneurial support was positively associated with entrepreneurial intention, aligning with prior international studies emphasizing the importance of entrepreneurial ecosystems and policy support in enhancing students' perceived feasibility of entrepreneurship (Elnadi and Gheith 2021; Gulzar and Fayaz 2023). Korea's government has implemented supportive measures for start‐ups and small‐medium enterprises (SMEs), such as the Act on Special Measures for the Promotion of Venture Businesses (S. Kim 2020) and the establishment of the Ministry of SMEs and Start‐ups (Kim et al. 2022). Yet awareness of such programs among young adults remains low (G. Kim 2018). Integrating information about these initiatives into university curricula and fostering partnerships between educational institutions and government agencies could bridge this knowledge gap (Kim and Yang 2022).

Overall, international studies have emphasized individual traits, social influence, educational support and entrepreneurial ecosystem factors in shaping entrepreneurial intention (Antončič and Auer Antončič 2023; Elnadi and Gheith 2021; Lingappa et al. 2020; Zhang et al. 2022). In this study, however, interpersonal and policy level contexts appeared more salient than individual dispositions among Korean nursing students. This pattern may reflect the Korean nursing education context, where hospital‐based employment is perceived as a stable career pathway and nurse‐led entrepreneurship remains relatively less visible.

Accordingly, nursing entrepreneurship education should provide concrete multilevel support. Nursing schools should implement peer‐supported activities, such as team‐based innovation projects, peer mentoring and student‐led idea‐sharing sessions; introduce family‐inclusive strategies that communicate the professional and social value of nurse‐led entrepreneurship; and link entrepreneurship curricula to clinical innovation, digital health, community‐based care, or healthcare service development. At the policy level, students should receive practical guidance on government start‐up funding, mentoring, incubation programs and healthcare innovation support systems. Collaboration among nursing schools, healthcare institutions, professional organizations and public agencies is needed to make entrepreneurship a visible and viable pathway for future nurses.

This study has limitations that should be considered when interpreting the findings. The cross‐sectional design limits causal interpretation, and the use of self‐reported data may have introduced response bias. Participants were recruited through convenience sampling via closed online nursing student communities, which may have introduced selection and self‐selection bias. More digitally engaged or entrepreneurship‐interested students may have been more likely to participate, potentially influencing participant characteristics and observed associations. Consequently, the sample may not be nationally representative, limiting generalizability to Korean nursing students, while its restriction to Korea may also limit transferability to other cultural and educational contexts.

In addition, because the survey was administered using Google Forms, the number of students who viewed the recruitment post, accessed the survey link, or started but did not submit the survey could not be determined. Therefore, the exact size of the accessible population, a denominator‐based response rate and a complete participant flow diagram with numbers at each recruitment stage could not be constructed.

Furthermore, the translated instruments underwent forward–backward translation and expert content validity review but were not pilot‐tested with target respondents, which may have limited assessment of item clarity, response burden and cultural relevance. Although items with very low standardized factor loadings were removed to improve measurement validity, AVE values for some constructs remained below the conventional 0.50 threshold, and this post hoc refinement may also limit comparability with studies using the original instruments; convergent validity for the affected constructs should therefore be interpreted with caution. Future studies should further validate the refined Korean versions of these scales among larger and more diverse samples of nursing students.

Despite these constraints, the current study provides a valuable foundation for comprehending the complex factors shaping entrepreneurial intention among nursing undergraduates. By addressing the interplay between individual, interpersonal, organizational and policy level influences, this research contributes to advancing entrepreneurial education and policy development tailored to the nursing profession.

## 5 Conclusion

Among Korean nursing students, entrepreneurial intention was primarily associated with interpersonal and policy level contexts rather than individual traits. Peer influence was the strongest explanatory variable, followed by family influence and perceived government entrepreneurial support. Notably, entrepreneurial intention was positively associated with perceived government support but negatively associated with favourable economic perceptions, a counterintuitive pattern likely reflecting a preference for secure, hospital‐based employment over entrepreneurial risk in Korea's competitive job market.

These findings suggest that fostering nursing students' entrepreneurial intention requires multilevel strategies that go beyond individual competency development. For nursing education and the broader nursing profession, peer‐supported learning, culturally appropriate family engagement and greater awareness of government support programs may help prepare future nurses to develop innovative services, technologies and care models. Overall, nursing entrepreneurship should be supported through coordinated efforts among educational institutions, families, professional communities and policymakers.

## Author Contributions

**Ji Young Lim:** conceptualization, methodology, validation, investigation, resources, data curation, project administration, funding acquisition, writing – review and editing, supervision. **Wonjung Noh:** conceptualization, investigation, methodology, validation, writing – review and editing. **Mohiniso Vohidova:** methodology, validation, writing – review and editing, writing – original draft. **Anna Lee:** conceptualization, methodology, validation, investigation, resources, formal analysis, data curation, writing – original draft, writing – review and editing, visualization.

## Funding

This work was supported by the National Research Foundation of Korea funded by the Korean government (No. RS‐2023‐00277435). However, this grant did not influence any aspects of the research, including its purpose, design, methods, results, or interpretation.

## Ethics Statement

This study was approved by the Yonsei University Health System IRB (no. 4–2024‐0561). Informed consent was obtained online from all participants prior to survey completion.

## Consent

This study does not contain any personal details, images, or videos that require consent for publication.

## Conflicts of Interest

The authors declare no conflicts of interest. Although a co‐author received a grant, it did not influence any aspects of the research, including its purpose, design, methods, results, or interpretation.

## Data Availability

The data that support the findings of this study are available on request from the corresponding author. The data are not publicly available due to privacy or ethical restrictions.
